# RF acceleration of ultracold electron bunches

**DOI:** 10.1063/4.0000200

**Published:** 2023-10-03

**Authors:** D. F. J. Nijhof, T. C. H. de Raadt, J. V. Huijts, J. G. H. Franssen, P. H. A. Mutsaers, O. J. Luiten

**Affiliations:** 1 Department of Applied Physics and Science Education, Coherence and Quantum Technology Group, Eindhoven University of Technology, P.O. Box 513, 5600 MB Eindhoven, The Netherlands; 2 Institute for Complex Molecular Systems, Eindhoven University of Technology, P.O. Box 513, 5600 MB Eindhoven, The Netherlands; 3 Doctor X Works BV, 5616 JC Eindhoven, The Netherlands

## Abstract

The ultrafast and ultracold electron source, based on laser cooling and trapping of atomic gas and its subsequent near-threshold two-step photoionization, is capable of generating electron bunches with a high transverse brightness at energies of roughly 10 keV. This paper investigates the possibility of increasing the range of applications of this source by accelerating the bunch using radio frequency electromagnetic fields. Bunch energies up to 35 keV are measured by analyzing the diffraction patterns generated from a mono-crystalline gold sample. It is found that the normalized transverse emittance is largely preserved during acceleration.

## I. INTRODUCTION

In recent years, the ultracold electron source (UCES) has been developed at the Eindhoven University of Technology to produce stable electron bunches at energies up to 10 keV with normalized transverse emittances on the order of a few nm · rad at repetition rates of 1 kHz.^1–8^

The bunches extracted from this source are created by two-step near-threshold photoionization of a laser-cooled and trapped rubidium gas. Their kinetic energy is limited by the static electric field strength in which the laser cooling and trapping occurs. This electric field strength is limited to ≈ 1 MV/m due to the broadening of the laser-cooling transition by breaking the *m_F_* degeneracy (Stark shift). In practice, this results in kinetic energies of ≤ 10 keV.^8,9^

Additional acceleration of these bunches is desirable not only because of the increased scope of applications like the investigation of thicker samples and/or sample environments, but also because of the reduced influence of the bunch's self-fields that are partially responsible for bunch quality degradation during free-space propagation.^10^ One way of achieving this is through the use of a resonant radio frequency (RF) cavity utilizing a transverse magnetic (TM) mode.^11^ In such a mode, the electric field is parallel to the propagation direction of the electron bunches, allowing for additional acceleration.

Such an RF cavity has been designed at the Coherence and Quantum Technology (CQT) group for the purpose of longitudinally compressing high charge electron bunches to sub-ps pulse durations.^12^ This cavity, operating at a frequency of 2.998 55 GHz (S-band) in the TM_010_ mode can also be used to accelerate ultracold electron bunches, even though it was not originally designed for this.

Work presented in this paper will first investigate the viability of this method through the particle tracking software general particle tracer (GPT).^13^ Data will then be presented, showing that ultracold bunches have been accelerated in this fashion and their final kinetic energy is determined through diffraction measurements on a mono-crystalline gold sample.^14^ Furthermore, analysis of the diffraction pattern will show that the electron bunch transverse beam quality is (largely) preserved during acceleration.

The structure of this paper is organized as follows: first, a brief overview of the UCES is presented in Sec. II. In Sec. III, the electron beamline and RF infrastructure are introduced. In Sec. IV, the viability of additional acceleration through an RF cavity will be examined by investigating the relevant bunch parameters via GPT simulations. Measurements are then discussed in Sec. V, where diffraction patterns generated from a mono-crystalline gold sample are analyzed to calculate the acquired kinetic energy of the bunches. Finally, in Sec. VI, additional analysis of the measured diffraction spots and results in estimations of the (transverse) normalized emittance of the accelerated electron bunches are presented.

Measurements show that bunches with an initial kinetic energy of 7.3 ± 0.1 keV are accelerated using an RF cavity to energies up to 35 keV with a transverse coherence length of 1–2 nm, easily resolving >40 diffraction peaks of a mono-crystalline Au sample. Transverse bunch quality estimations based on simulations and experimental data further indicate a transverse normalized emittance of ≤ 10 nm · rad after additional acceleration. On the basis of the experiments presented in this work, a design study of a dedicated RF accelerator capable of accelerating the bunches to 100 keV using a commercially available solid-state RF amplifier was done.^15^

## II. THE ULTRACOLD ELECTRON SOURCE

The electron source used to create the ultracold electron bunches described in this paper works through the laser-cooling and trapping and subsequent (two-step) photoionization of an ^85^Rb atomic gas. The predecessor of this source consisted of three pairs of mutually perpendicular laser beams.^16,17^ High-quality diffraction patterns were obtained with this source and electron temperatures of <30 K were found for femtosecond photoionization bunch generation.^4,6,18^

**FIG. 1.**
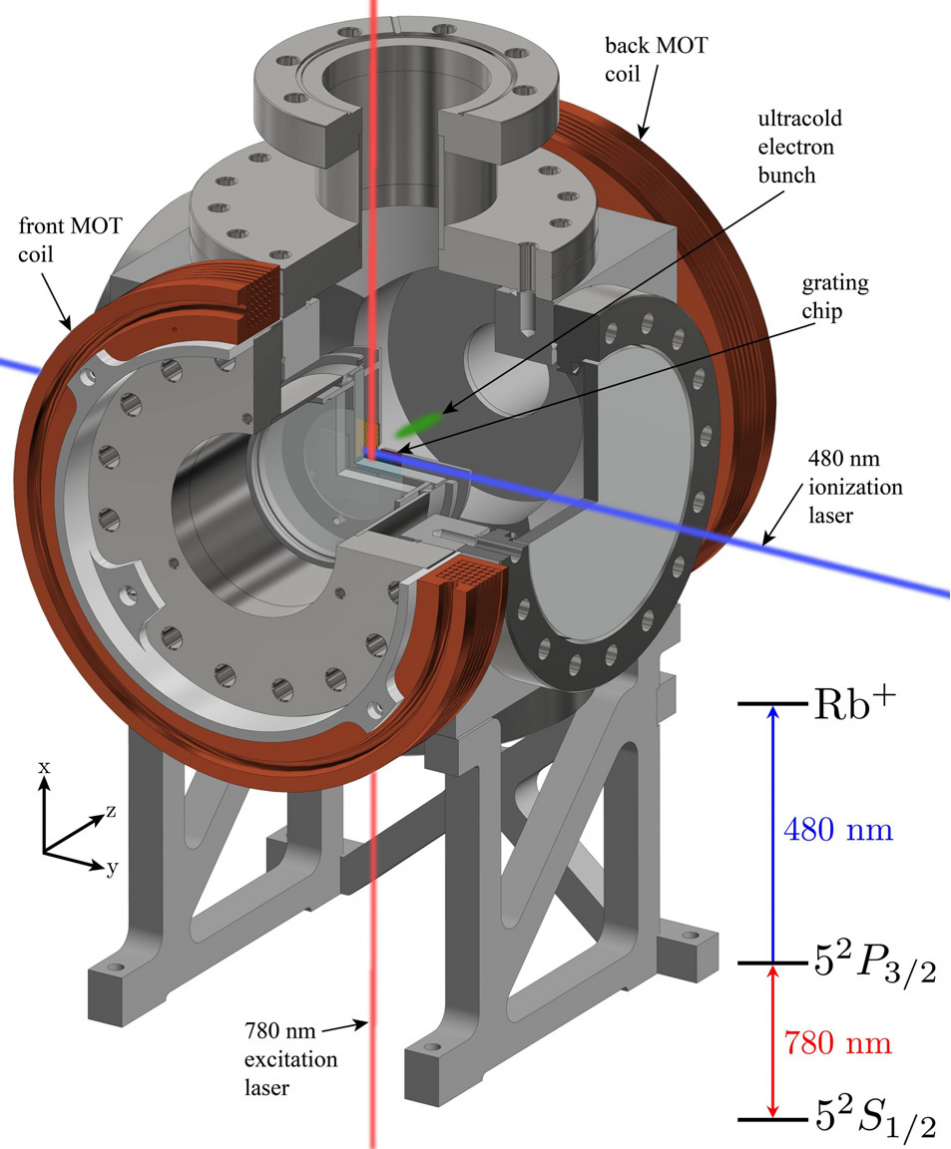
Schematic representation of the UCES showing the excitation and ionization laser creating an electron bunch (green), which is accelerated in the $\hat{z}$-direction.

The current ultracold electron source, shown schematically in Fig. 1, retains the same operational principle: rubidium gas is laser-cooled and trapped and electron bunches are extracted using a static field through near-threshold femtosecond photoionization. This section will give a brief description of the source as is relevant for this work.

Instead of using three pairs of mutually perpendicular laser beams to realize laser-cooling and trapping, a single circularly polarized red-detuned laser beam (frequency stabilized to the cooling transition $5^{2}S_{1/2}F=3\to 5^{2}P_{3/2}F=4$ of ^85^Rb) is used in conjunction with a grating chip to achieve this^19^ (for a more detailed description, the reader is referred to work by Franssen^8^ and van Ninhuijs^20^).

To create electron bunches, the trapping laser is turned off for a few *μ*s after which the rubidium atoms are again excited using a 780 nm laser to the $5^{2}P_{3/2}F=4$ state and subsequently ionized by a wavelength tunable 480 nm fs laser pulse, propagating perpendicular to the excitation laser, see Fig. 1. The wavelength tunability of the ionization laser enables the generation of electrons with very low excess energies. The laser cooling and trapping, excitation, and ionization are done in a static extraction field of ≈ 1 MV/m at the region of ionization, which results in ≤ 10 keV electron bunches when the transparent cathode is kept at −20 kV.

Typically, electron bunches are created with an initial rms source size of 30 *μ*m in all directions. This typically results in rms normalized emittances of 0.4–2.8 nm · rad, corresponding to 10 K electron temperatures based on GPT simulations.^8^ Recently, pondermotive measurements have been performed in the self-compression point, resulting in rms bunch lengths of 735 ± 7 fs.^21^

## III. EXPERIMENTAL DESIGN

### A. Electron beamline

**FIG. 2.**
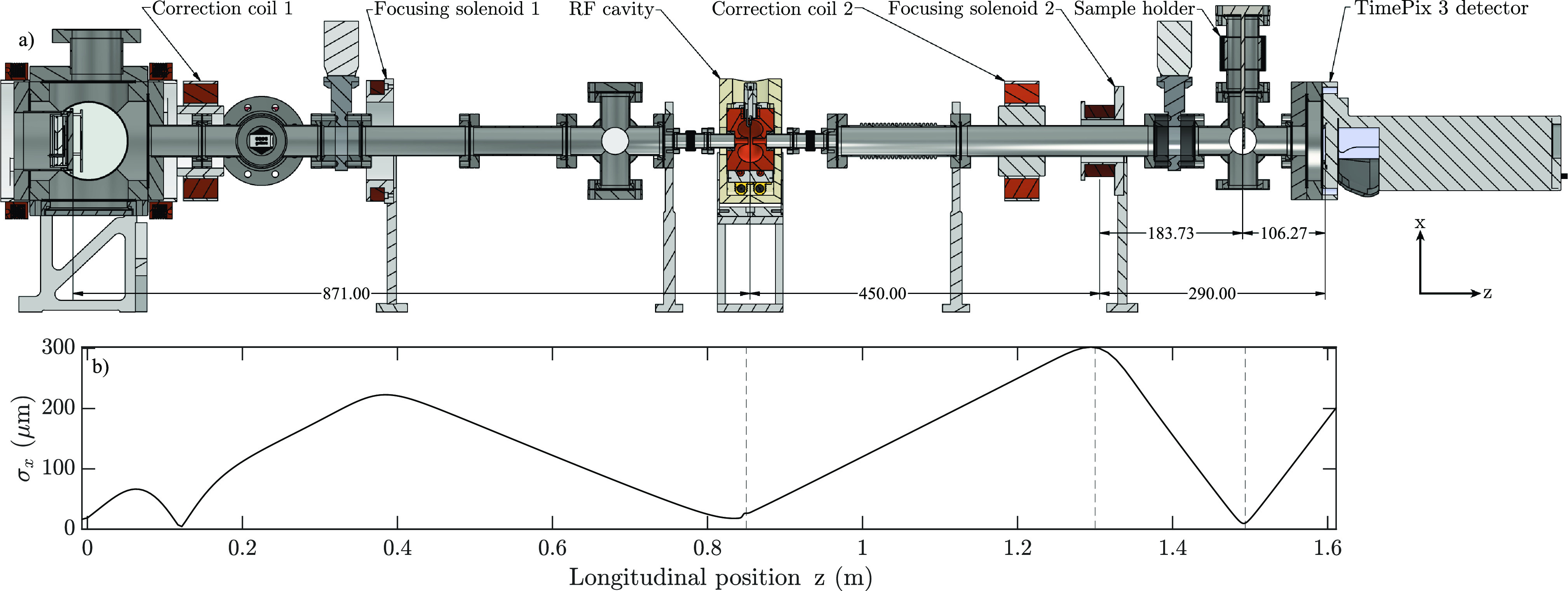
(a) Schematic representation of the UCES beamline showing the main components (from left to right): the UCES, RF cavity, focusing solenoid, Au sample holder, and the TimePix 3 detector (units in mm). (b) The rms transverse bunch size as a function of the longitudinal position. The RF cavity, focusing solenoid, and Au sample locations are indicated by dashed vertical lines.

A schematic representation of the beamline used during the experiment is given in Fig. 2(a), where the UCES is shown, including correction and focusing coils, the TM_010_ cavity, the sample holder which holds the Au sample, and the detector. The detector used in this work is an event-driven TimePix 3, which is capable of capturing diffraction patterns without the need to block the high intensity central peak.^22^ Figure 2(b) shows the typical evolution of the transverse rms beam size throughout the beamline with the locations of key components indicated by vertically dashed lines.

**FIG. 3.**
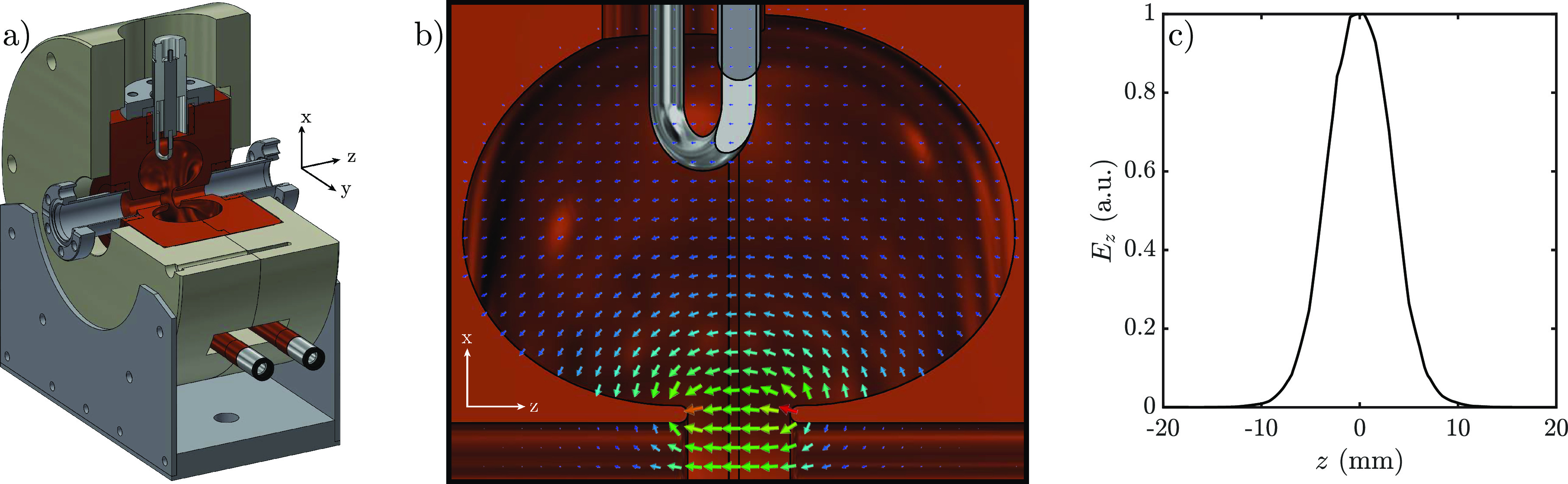
(a) A schematic representation of the TM_010_ cavity, (b) a cross-sectional view of the cavity's interior with the arrows indicating the electric field vectors in the *x*–*z* plane [colors ranging from blue (weak) to red (strong)], and (c) the on-axis longitudinal electric field profile.

The cavity used in this work is shown in Fig. 3(a) where the copper structure, the thermally insulating material encasing it, the coupling antenna, and the water-cooling connections are visible. Figure 3(b) shows the top-half of the copper structure in the *x*–*z* plane where the arrows indicate the direction and magnitude of the electric field. Enhancement of the on-axis field strength by the nosecones can clearly be seen. Nose cones increase the accelerating field strength along the optical axis in the accelerator without requiring more input power, increasing its efficiency. Figure 3(c) shows the on-axis longitudinal field strength *E_z_* based on cst microwave studio^23^ simulations shown in Fig. 3(b).

### B. RF infrastructure

Additional acceleration of the ultracold electron bunches is realized through the use of an RF cavity. For this to happen, energy needs to be stored in the cavity in the form of electromagnetic fields. In this work, a pulsed operation of the accelerating cavity is used: RF pulses with a duration of 10 *μ*s were used to fill the cavity with the required energy. The generation of the RF signal is realized as follows: a Ti:Saph-based mode-locked oscillator (Coherent MANTIS) is used as the master clock of the experiment, operating at 74.9625 MHz. This signal is used to synchronize a 2.998 55 GHz electronic oscillator (40th harmonic) to the laser system.^24^

The phase-stabilized 2.998 55 GHz continuous-wave (CW) signal is used as the input for the generation of the 10 *μ*s RF pulses. The cavity is designed to operate at $f_{0}=2.998\;55$ GHz with a measured unloaded quality factor of $Q_{0}=8960$.^25^ More information on the generation of the RF pulse and the characterization of the cavity can be found in Ref. 28.

## IV. PARTICLE TRACKING SIMULATIONS

general particle tracer^13^ simulations have been done with realistic beamline parameters to investigate the bunch's behavior. Throughout the experiments, the applied potential to the static accelerator was set to −16 kV and the laser-cooled and trapped atom cloud was created approximately 7 mm in front of the grating surface. The excitation and ionization lasers had rms spot sizes of 16.3 ± 1.2 and 15.3 ± 1.2 *μ*m, respectively, creating bunches with a charge on the order of 0.1 fC. An electron temperature of 10 K is assumed for these simulations.^8^

**FIG. 4.**
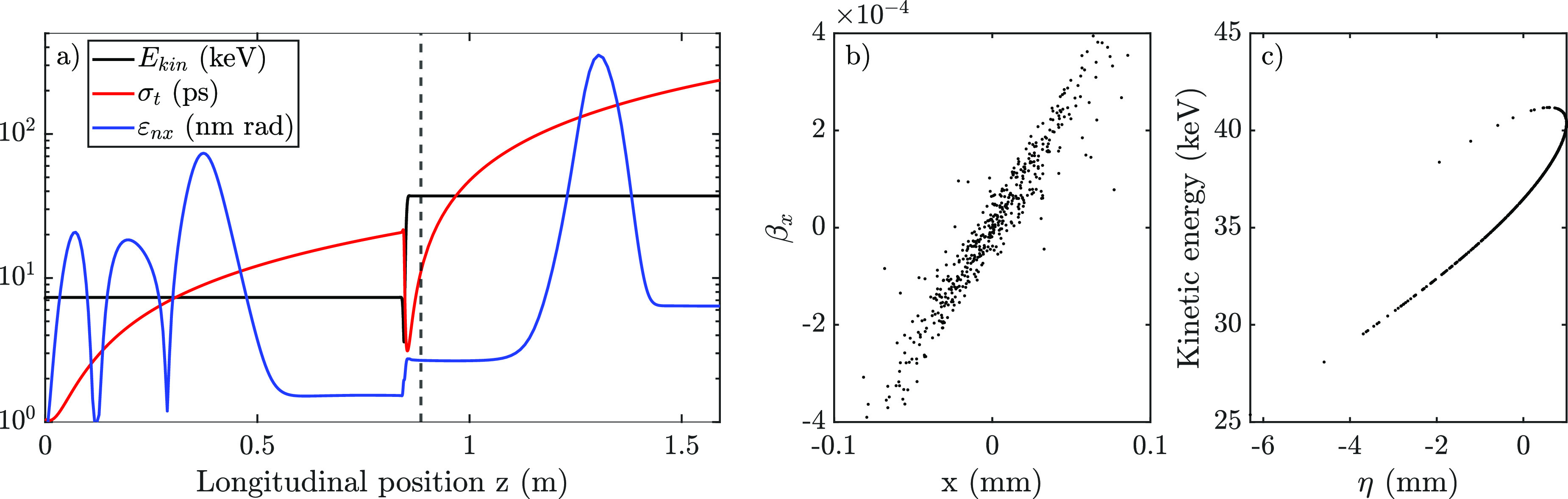
(a) GPT simulations of a 0.1 fC bunch showing the kinetic energy (black), rms temporal length (red), and the normalized transverse emittance in the *x*-direction (blue) as a function of the longitudinal position, (b) the transverse phase space (*x*-*β_x_*), and (c) the kinetic energy as a function of the relative longitudinal coordinate $\eta \equiv z-\bar{z}$, evaluated at the position of the dashed line in (a).

These parameters result in bunches leaving the UCES at approximately 7.3 keV, based on realistic field maps, as shown in Fig. 4(a), along with the rms temporal length *σ_t_* and normalized transverse emittance $\varepsilon_{nx}$, plotted as a function of the longitudinal position *z*.

As the bunches drift toward the accelerator, they stretch in the longitudinal direction after exiting the source due to the energy spread. This energy spread is directly related to the dimensions of the overlap of the excitation and ionization laser in the trapping region; electrons created at the back of the bunch are accelerated to higher energies compared to electrons created at the front. This results in a longitudinal self-compression point just behind the exit of the source, after which the bunch longitudinally diverges.

The bunch arrives at the RF accelerator with an rms pulse length of 21.6 ps. The pulse length of the bunch, combined with a low kinetic energy and a 333 ps period of the RF accelerator, results in non-homogeneous acceleration of the bunch (this is mainly due to the long transit time of the bunches in the cavity, which was originally designed for 100 keV bunches^12^). The transverse/longitudinal phase space distributions of the bunch exiting the cavity is shown in Figs. 4(b) and 4(c), where *η* represents the relative longitudinal position of each electron in the bunch with respect to their average longitudinal position $\bar{z}$. These bunch distributions have been calculated just behind the exit of the accelerator, indicated by the dashed line in Fig. 4(a). An average kinetic energy on the order of 40 keV is expected when a forward power of 500 W is supplied to the accelerator.

During the drift to the accelerator, the normalized transverse emittance grows to 1.5 nm · rad; during acceleration, it increases to 2.7 nm · rad and becomes 6.4 nm · rad after being strongly focused on the sample.

## V. KINETIC ENERGY MEASUREMENTS

### A. DC acceleration

First, measurements are conducted without delivering power to the RF accelerator, i.e., only DC acceleration to approximately 7 keV. The bunch is focused on a mono-crystalline Au sample positioned at a distance of 106 mm from the detector. The crystal structure of Au is known to be face-centered cubic, with a lattice parameter of 4.07 Å,^26^ the approximately 11 nm thick crystal is grown in a (100) orientation through chemical vapor deposition, giving familiar lattice spacings.^14^

An expression for the longitudinal momentum of the bunch as a function of the separation between the first order diffracted peaks and the zeroth order peak (henceforth referred to as the direct beam) can be found by substituting the expression for the de Broglie wavelength in Bragg's diffraction law and rewriting in terms of $m_{e}c\gamma \beta$ as follows:

$$m_{e}c\gamma \beta =\frac{nh}{2d_{\text{hkl}}\;\text{sin}\;\theta_{B}},$$ (1)

where *d_hkl_* is the spacing between parallel lattice planes with Miller indices *hkl*, *θ_B_* the Bragg angle, *n* the order of diffraction, *h* the Planck's constant, *m_e_* the electron rest mass, *c* the speed of light, $\gamma =\frac{1}{\sqrt{1-\beta^{2}}}$ the Lorentz factor, and $\beta =\frac{v}{c}$ the normalized bunch velocity.

An overview of the relevant crystal parameters of the gold sample is given in Table I. Here, the peak positions are given by $q_{\text{hkl}}=\frac{2\pi }{a}\sqrt{h^{2}+k^{2}+l^{2}}$, and the spacing between parallel lattice planes is given by $d_{\text{hkl}}=\frac{2\pi }{q_{\text{hkl}}}$.

**TABLE I.** Au crystal parameters: the Miller indices *hkl*, the peak positions *q_hkl_*, and the parallel lattice spacings *d_hkl_.*

*hkl*	*q_hkl_* (nm^–1^)	*d_hkl_* (pm)
2 0 0	30.81	203.91
2 2 0	43.58	144.19
3 1 1	51.10	122.96
4 0 0	61.63	101.95
4 2 0	68.90	91.19
5 1 1	80.06	78.49
4 4 0	87.45	72.09
6 0 0	92.44	67.97
6 2 0	97.44	64.48

**FIG. 5.**
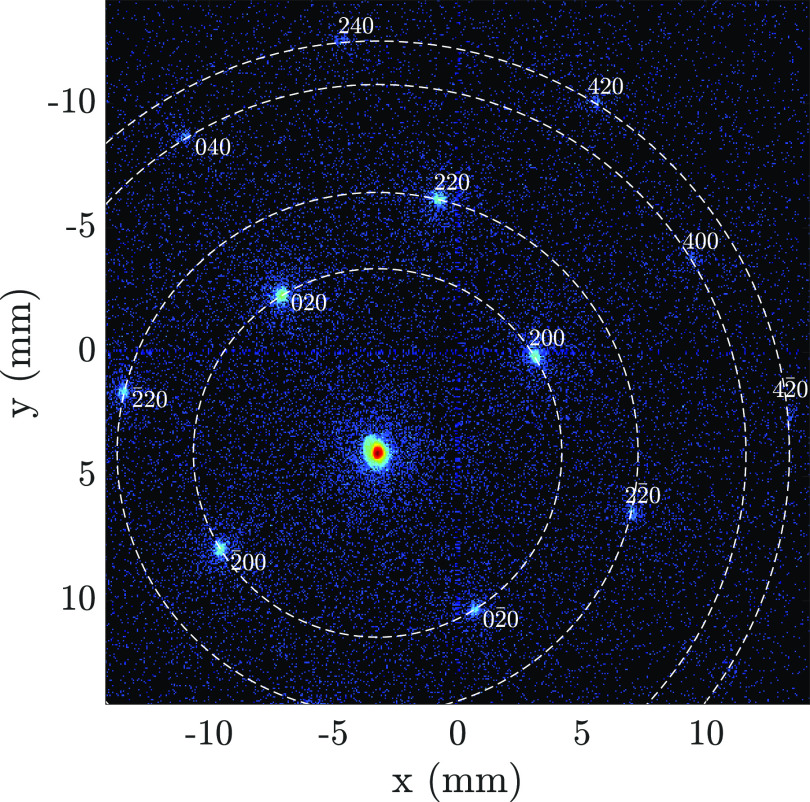
Detector image of the diffraction pattern of a mono-crystalline Au sample produced by 1000 electron bunches extracted from the UCES without additional RF acceleration. The numbers indicate the Miller indices *hkl* corresponding to the observed diffraction peak with the dashed line indicating the diffraction rings that would be obtained if the sample had been polycrystalline.

With the distance between the gold sample and the detector plane known, the kinetic energy can be calculated from the measured separation between the direct beam and first order diffracted spots. Figure 5 shows the summed diffraction pattern consisting of ten separate measurements, each containing 100 shots, obtained at a repetition frequency of 1 kHz. The color scale is logarithmic to increase the visibility of the low intensity peaks. Here, the first order diffraction peaks are clearly visible, indicated by their corresponding *hkl* values in white numbers. From this measurement, the initial kinetic energy of the ultracold electron bunches is determined to be 7.3 ± 0.1 keV through Eq. (1), corresponding to the expected initial bunch energy based on GPT simulations with realistic parameters.

### B. RF acceleration

**FIG. 6.**
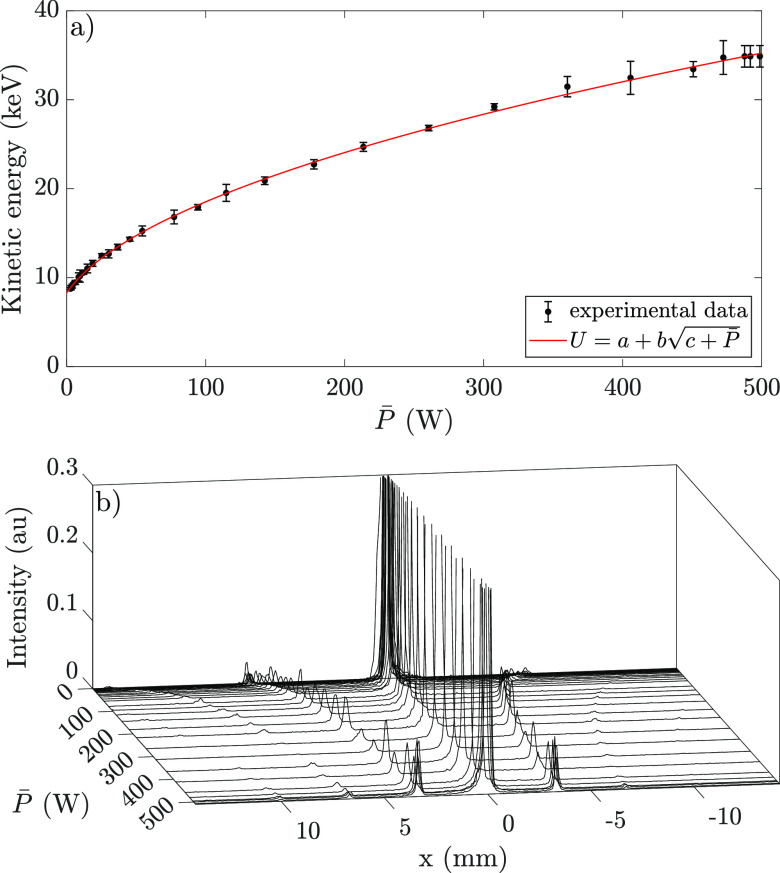
(a) The measured kinetic energy of the accelerated bunches as a function of the RF power supplied to the cavity (black data points) fitted with a basic square-root function (solid red), with *a* = 3.80, *b* = 1.38, and *c* = 9.14. (b) 3D plot showing a line-out profile (along the center) of the detector image as a function of the power supplied to the accelerator.

Power is now supplied to the RF accelerator. The results of the measurements are presented in Fig. 6(a), which shows the determined kinetic energy of the electron bunches as a function of the power delivered to the accelerator. During this measurement series, the seed RF power is increased in steps of 1 dB. The data points represent the average of the determined kinetic energies of the combined 020 and $0\bar{2}0$ diffraction spots, the error bars give the standard deviation in the obtained energy from those spots. In red, a square-root fit shows excellent agreement with the measured data.

Figure 6(b) shows the normalized line-out profile (along the horizontal axis) of detector images displayed in a 3D plot as a function of the power supplied to the accelerator. Here, it can clearly be seen that diffraction spots move toward the direct beam as the power delivered to the RF cavity increases.

**FIG. 7.**
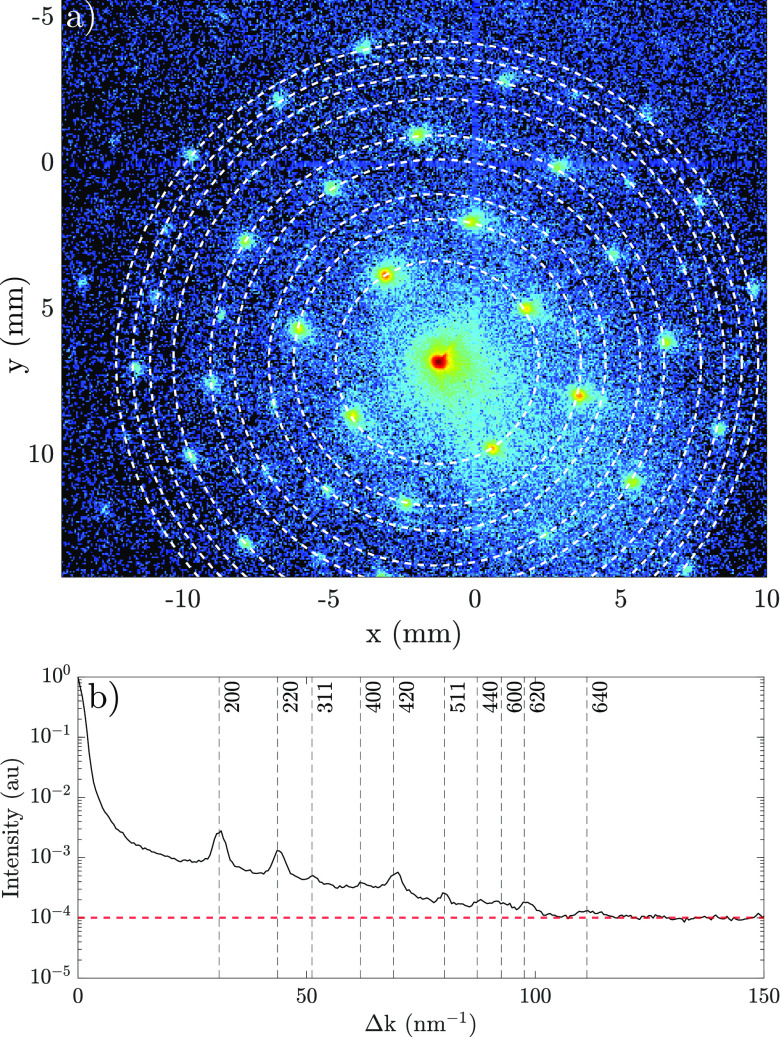
(a) Diffraction pattern collected on the Cheetah T3 detector, generated by $3\times 10^{3}$ bunches with an energy of 32.6 keV (412 W supplied to the accelerator) on Au; the dashed rings indicate the groups of Miller indices listed in Table I. (b) The corresponding Δk-space showing the azimuthally averaged intensity with the theoretically calculated reciprocal lattice planes *q_hkl_* indicated by black dashed lines; the red-dashed line indicates the noise floor level.

Figure 7(a) shows a diffraction pattern obtained with 32.6 ± 0.1 keV electron bunches (3000 bunches in total). Many more diffraction peaks have become visible on the detector compared to the image shown in Fig. 5. Figure 7(b) shows the azimuthally averaged intensity plot of the corresponding figure above it. The dashed lines correspond to the diffraction peak groups that were obtained from theory, see *q_hkl_* in Table I, Δk is obtained through $\Delta k=2\left|k_{0}\right|\;\text{sin}\;\theta_{B}$ with data from the detector image.

## VI. BUNCH QUALITY

From the diffraction patterns obtained in Sec. V, the transverse coherence length *L_T_* of the electron bunches at the sample plane can be calculated via

$$L_{T}\equiv \frac{\hbar }{\sigma_{p_{x}}}=\frac{\lambda_{B}}{2\pi \sigma_{\theta }},$$ (2)

where $\lambda_{B}=\frac{h}{m_{e}c\gamma \beta }$ is the de Broglie wavelength, and $\sigma_{\theta }$ is the rms angular divergence of the electron bunch at the sample plane. During the diffraction experiments, the transverse beam waist is repositioned on the Au sample, which is done by varying the current through the focusing solenoid, each time the power supplied to the RF cavity is increased.

This allows analysis of the detector image under the assumption that the rms angular divergence $\sigma_{\theta }$ dominates the diffracted spot sizes.^18^ For this work, this criterion becomes $\sigma_{\theta }\gg \frac{\sigma_{\text{sample}}}{D}\approx 0.18$ mrad where $\sigma_{\text{sample}}$ is the rms spot size on the Au sample, and *D* the separation between the Au sample and the detector plane.

Equation (2) can be re-written by substituting the diffraction angle obtained from Bragg's law: $\gamma \beta \approx \frac{h}{2d_{\text{hkl}}\theta_{B}m_{e}c}$ under the paraxial approximation, here $d_{\text{hkl}}\approx 2.04$Å is the shortest distance between two crystal planes of Au for which diffraction occurs (see Table I). The transverse coherence length can then be expressed as $L_{T}\approx \frac{d_{\text{hkl}}\theta_{B}}{\pi \sigma_{\theta }}$. Finally, the substitutions $\theta_{B}\approx \frac{s}{2D}$, with *s* being the separation between the direct beam and the (200) diffraction spots, and $\sigma_{\theta }\approx \frac{\sigma_{d}}{D}$, with *σ_d_* being the rms spot-size of the diffracted spots on the detector, are made, resulting in the following expression for the transverse coherence length:

$$L_{T}\approx \frac{d_{\text{hkl}}s}{2\pi \sigma_{d}}.$$ (3)

With this expression, *L_T_* can be determined by analyzing portions of diffraction patterns like the one shown in Fig. 7(a). In this configuration, the analysis can be extended to estimate the normalized transverse emittance of the bunch at the sample plane,

$$\varepsilon_{n}=\frac{ƛ}{C_{T}},$$ (4)

where $ƛ=\frac{\hbar }{m_{e}c}\approx 0.39$ pm is the reduced Compton wavelength, and $C_{T}=\frac{L_{T}}{\sigma_{\text{sample}}}$ is the relative transverse coherence at the sample. Note here that the rms transverse spot size on the gold sample is obtained through GPT simulations based on experimental settings; thus, the obtained values for the transverse emittance and relative coherence are estimations based on both simulations and experimental data.

**FIG. 8.**
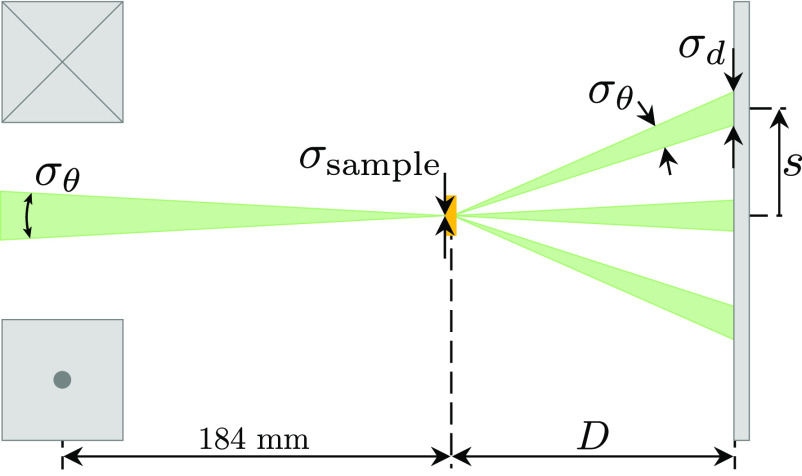
A schematic representation of the diffraction experiment showing relevant parameters used in the determination of the transverse coherence length *L_T_*. The gray rectangles on the left represent the focusing solenoid used to focus the electron beam (green) on the gold sample (orange), $\sigma_{\text{sample}}$ represents the rms spot size on the gold sample, $\sigma_{\theta }$ the rms divergence angle of the beam, *σ_d_* the rms spot size of the diffracted spots on the detector, *s* the distance between the direct beam and the (200) diffracted spots, and *D* is the separation between the gold sample and the detector (gray).

A schematic representation of the diffraction experiment is shown in Fig. 8 where the incident beam (green) is focused by the focusing solenoid (gray boxes) to a spot size $\sigma_{\text{sample}}$ onto the gold target (orange). The rms angular divergence $\sigma_{\theta }$ projects a diffracted beamlet with rms size *σ_d_* at a distance *s* from the direct beam to register on the detector (gray slate) separated from the sample by a distance *D*.

**FIG. 9.**
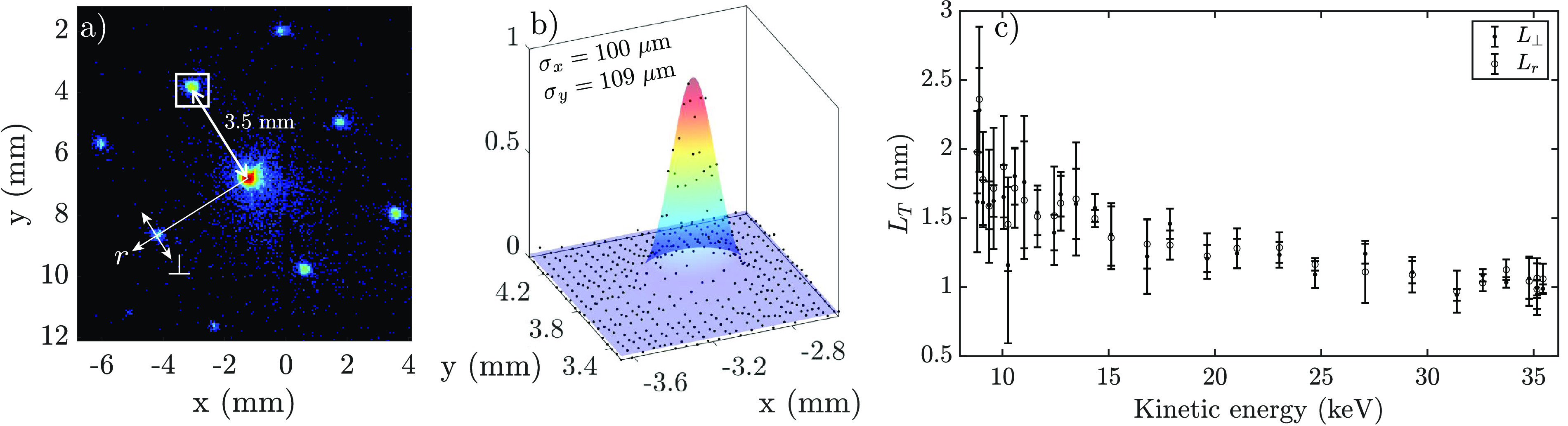
Routine for determining the transverse coherence length: (a) a portion of the diffraction pattern presented in Fig. 7, showing the separation between the unscattered electrons and the first order diffraction peak, (b) a two-dimensional Gaussian surface fit through the (200) diffraction spot highlighted by the white box, (c) and the resulting calculated transverse coherence lengths in the *x-* and *y*-direction, as calculated by Eq. (3), as a function of the calculated bunch energies according to Eq. (1).

The transverse coherence length is now calculated for every composite diffraction pattern (consisting of 1000 shots taken during ten 0.1 s exposures), of which three are collected per RF power setting. First, the separation *s* between the diffraction spots and the direct beam is determined as is shown in Fig. 9(a). These spots are subsequently fitted with a 2D Gaussian intensity profile, from which the rms widths $\sigma_{⊥}$ and *σ_r_* are determined [Fig. 9(b)]. This is done for the 020, 200, and $0\bar{2}0$ diffraction spots (see Fig. 5).

As the energy spread affects the diffracted bunch profiles in the radial direction *r*, the bunch dimensions whose directions are perpendicular ( ⊥) to the radial coordinate are treated separately; these directions are indicated in Fig. 9(a). From these data, it is found that the average rms spot sizes of the diffracted beamlets on the detector for all RF attenuation settings are $\sigma_{⊥}=118\pm 13$ and $\sigma_{r}=118\pm 18\;\mu$m, respectively. This results in a corresponding average rms angular spread on the sample of 1.1 mrad in both the radial and transverse direction, satisfying the requirement that $\sigma_{\theta }\gg 0.18$ mrad.

From these data, the transverse coherence lengths are calculated, shown in Fig. 9(c). The error bars give the standard deviation in these data points per the average determined kinetic energy. It is found that as the bunches are accelerated to higher energies, the transverse coherence length converges to $L_{T}\approx 1$ nm.

As mentioned earlier, the transverse waist of the electron beam is made to coincide with the sample as closely as possible by varying the current to the focusing solenoid. The somewhat larger values for *L_T_* at smaller bunch energies can be attributed to the positioning of the transverse waist during the experiment. In Ref. 28, it is shown that lower energetic bunches have a transverse waist before the Au sample plane, according to GPT simulations based on inputs from the experiment. This results in a larger drift distance to the detector, and subsequently a larger value in the numerator of Eq. (3).

**FIG. 10.**
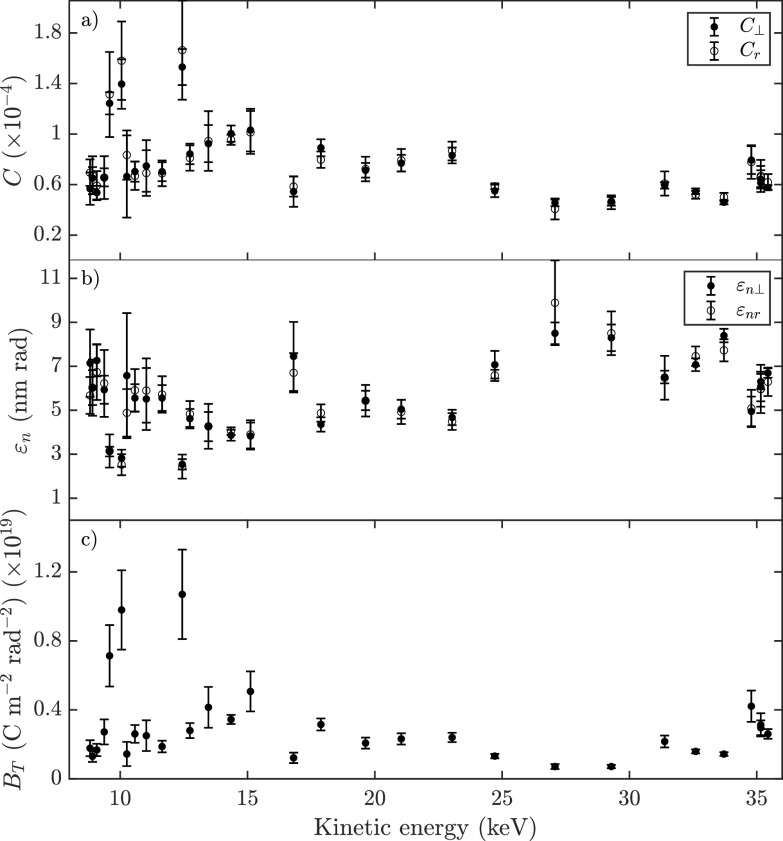
Bunch parameters based on a combination of the measured transverse coherence length and simulated bunch properties, showing (a) the relative transverse coherence, (b) the normalized transverse emittance, and (c) the reduced transverse brightness.

The relative coherence at the location of the Au sample is shown in Fig. 10(a) as a function of the measured kinetic energy of the bunches. The rms spot size on the Au sample is obtained through GPT simulations and was found to be 19±6 μm when averaged over all different powers supplied to the RF accelerator, taking into account the varying current supplied to the focusing solenoid.

Figure 10(b) shows the normalized transverse emittance as calculated by Eq. (4). The initial normalized “thermal” emittance of the bunch at the source is determined by the electron temperature and the volume from which they are extracted. The rms transverse spot sizes of the excitation and ionization laser were measured to be $\sigma_{0}\approx 15\;\mu$m. These lasers cross perpendicularly, which defines the ionization “overlap” volume and thus the source size. On a theoretical basis, a perfect overlap between the two lasers will result in a thermal emittance of $\varepsilon_{n,\text{thermal}}=\sigma_{0}\sqrt{\frac{k_{b}T}{m_{e}c^{2}}}\approx 0.6$ nm · rad, assuming again 10 K electrons.^8^

The measured normalized emittance at the detector is, however, an order of magnitude larger. To explain this increase in the normalized emittance, detailed charged particle tracking simulations have been performed, taking into account realistic fields of the DC and RF accelerators, the charged particle optics, and Coulomb interactions between individual particles.^13^ Simulations show that the normalized emittance already increases in the first few millimeters after creation of the bunch, mainly due to the Coulombic self-fields of the bunch. Additional increase in the emittance can occur due to non-zero magnetic fields experienced by the bunch during creation in the MOT^27^ and due to non-linearities in the DC electric field. All these contributions result in a normalized transverse emittance of 1.5 nm · rad prior to entering the RF accelerator. With an optimized transverse bunch size and injection at an optimal initial RF phase, the normalized emittance grows to approximately 2.7 nm · rad during RF acceleration.

Further growth of the normalized emittance is attributed to a sub-optimal injection in the accelerator by e.g., misalignment. Simulations have been used to quantify the effect of misalignments by rotating the cavity about the *x*-axis, i.e., in the *y*–*z* plane [see Fig. 3(a)], resulting in an emittance increase of 4% when rotating the cavity 1°, and up to 50% at rotations of 5°.

Finally, the reduced transverse brightness $B_{T}=\frac{Q}{\varepsilon_{nr}\varepsilon_{n⊥}}$ is plotted in Fig. 10(c). The average amount of charge per RF setting reaching the detector is measured by the detector itself (see Ref. 28). With the normalized transverse emittances determined via Eq. (4), the reduced transverse brightness is found to be approximately constant, especially considering the relatively large error bars found for higher values of *B_T_*.

## VII. CONCLUSIONS AND OUTLOOK

Ultracold electron bunches produced through photoionization of laser-cooled atomic gas and extracted by a static field to 7.3 ± 0.1 keV have been accelerated to higher energies through the use of a resonant 2.998 55 GHz RF cavity operating in the TM_010_ mode. Measurements of electron diffraction on a single-crystal gold target have shown electron-bunch energies up to 35 keV, corresponding well with expectations obtained from detailed particle tracking simulations.

Acceleration to even higher kinetic energies can be easily realized with commercially available high power solid-state amplifiers and a dedicated RF accelerator structure.^15^ Incorporation of a bunching-cavity prior to accelerating the bunches should result in higher longitudinal and transverse coherence of the electron bunches and is desirable for further experiments like protein crystallography and the injection of ultracold electron bunches in high-gradient LINACs to reach MeV bunch energies.

The quality of the individual diffraction patterns implies that transverse bunch quality is largely preserved as the kinetic energy is tuned by varying the RF power to the accelerator. Transverse coherence lengths are found to be 1–2 nm when the bunch is accelerated to 35 keV. Combining the measured values for *L_T_* with particle tracking simulations resulted, under various assumptions, in estimated normalized emittances of ≤ 10 nm · rad. This value is relatively large compared to earlier measurements^8^ and is attributed to sub-optimal injection of the electron bunches in the accelerator.

## Data Availability

The data that support the findings of this study are available from the corresponding author upon reasonable request.
